# Replenishing Hope: The Time for Country-Led Integration Is Now

**DOI:** 10.34172/ijhpm.9284

**Published:** 2025-12-27

**Authors:** Nicola Watt, Diana Yousef, Alan Brooks, Elizabeth Juma, Kristin Saarlas, Kazim Sanikullah, Magid Al-Gunaid, Michael Byrne, Melanie Renshaw, Simon Bland, Ngozi A. Erondu

**Affiliations:** ^1^Deutsche Gesellschaft für Internationale Zusammenarbeit, Bonn, Germany.; ^2^Global Institute for Disease Elimination, Abu Dhabi, UAE.; ^3^Bridges to Development, Geneva, Switzerland.; ^4^World Health Organization Africa Regional Office, Brazzaville, Congo.; ^5^Task Force for Global Health, Decatur, GA, USA.; ^6^World Health Organization Western Pacific Regional Office, Manila, Philippines.; ^7^Eastern Mediterranean Public Health Network, Amman, Jordan.; ^8^The Global Fund to Fight AIDS, Tuberculosis and Malaria, Geneva, Switzerland.; ^9^African Leaders Malaria Alliance, Dar es Salaam, Tanzania.

## Time to Integrate

 There are just five years to go to reach the Sustainable Development Goals, the World Health Organization (WHO) Neglected Tropical Diseases (NTDs) Roadmap 2030, the 2016-2030 Global Technical Strategy for Malaria, and the Immunization Agenda 2030. These goals have become even more challenging for many low- and middle-income countries in the context of recent seismic shifts in the funding landscape for global health. At the same time, there is new momentum, including through the Lusaka Agenda and other ongoing processes, behind decade-old efforts to increase alignment between disease-specific global health initiatives and programs.^1,2^ Such initiatives risk competition for engagement from local health workers and communities and pose structural barriers to integration – which, under the right circumstances, seems to offer potential to achieve more sustainable impact with limited resources.^3,4^ In this viewpoint from the Integration Working Group, we use examples from integration of NTDs to argue that the time for integration is now. We call for governments to push for integrated planning – including across sectors – and for the international community to incentivise integration and support efforts to consolidate knowledge and best practice.

## Understanding Integration of Health Programs

 Integration is not a new concept and is a key focus in many technical strategies aligned with the above agendas. There is no single definition^[1]^, which can lead to unnecessary time lost in arguing over the terminology (“integration” is sometimes used interchangeably with “mainstreaming”) or the “right” way to integrate. There is literature across multiple fields studying the conditions which make integration most likely to result in benefit: a rapid literature review of publications on “neglected tropical disease” (NTD) “AND” “integration” listed on PubMed (using the MeSH subheading “prevention and control”) identified 135 separate publications since 2013 (ten years preceding the Group’s formation). These and other relevant literature (including grey literature) dating back to 1998 have informed the deliberations of the Group (Supplementary file 1). Multiple frameworks exist (Table), illustrating that integration is possible between different “domains” (policies, activities or organisations), at different levels (from local to global), and to different degrees (from information exchange to co-implementation).^5^ In the disease elimination community, integration is characterized by functionally coordinating certain aspects of disease control implementation across disease programs to reduce fragmentation, maximize resources, and increase equity in health care access.^4,6^

**Table:** A Typology of Integration^3,5^

**Typology of Integration**	
Domains	Across disease programs, whether clinically related or unrelated diseases Between vertical (disease-specific) and horizontal (system-wide) programs Across public health programs and health service interventions Across health and other sectors
Levels	Community, district, national, regional, global
Degree	From information exchange, through coordination to joint planning and implementation

 Integration is therefore not a one-size-fits-all solution. It aligns with the momentum towards stronger primary health care and universal health coverage, embracing the aims of leaving no one behind whilst aiming to increase quality and sustainability. It would be naïve, however, to believe that integration attempts always result in increased efficiency and better outcomes, or to think it can succeed without upfront investment. There is plentiful evidence about the potential challenges – aligning programs that might have started at different times with different aims; overcoming the additional complexity that working together inevitably demands, and inflexible funding – but also methods to help overcome them, including effective identification and communication of common benefits (“win-wins”).^7^ Successful integration necessitates a multifaceted strategy involving robust advocacy and leadership, accessible technical (including human) resources, and stakeholder collaboration.^3,7^

## An Established Benefit: Demonstrating Effectiveness Through Integration of Neglected Tropical Diseases

 Affecting 1.7 billion people, NTDs are closely linked to poverty, development, and environmental factors. Efforts to ringfence sufficient funding, and reliance on donated medicines have resulted in the establishment of vertical programs, but there is a compelling case against these approaches: research from the Health Campaign Effectiveness Coalition synthesising results from multiple efforts to integrate health campaigns found that integration did not compromise the primary campaign’s effectiveness, maintained high coverage for both interventions, and added value through co-delivery of treatments (eg, ivermectin for onchocerciasis and albendazole for soil transmitted helminths) and vaccines (eg, measles and meningitis). Integrated health campaigns can enhance community acceptability, provide opportunities to identify and refer zero-dose children, and be cost-effective.^8^

 More systematically, there are now countless examples where investing in integrating NTD management – not just across campaigns – is yielding significant improvements. Based on the deliberations of the Integration Working Group, including presentations by group members to the Conference in Public Health in Africa in 2023, we highlight a few of these examples here, to illustrate the diversity of approaches and starting points.

###  1. Cross-sectoral Integration – Zambia One Health^9^

 Zambia has successfully applied the One-Health approach to address taeniasis and schistosomiasis multi-drug administration, with cross-sectoral collaboration among diverse sectors to devise joint intervention strategies, including human health, veterinary/agriculture, and environmental ministries as well as the education sector. These strategies encompass coordinated efforts in mass drug administration, vector control, health education, and the mitigation of environmental factors contributing to the diseases. This collaborative effort ensures comprehensive coverage, ranging from 90% to 100%. Reported success factors including the engagement of academia to help establish surveillance systems for monitoring diseases in both human and animal populations, and the engagement of communities, civic leaders and community health workers in disseminating information on preventive measures, emphasizing the importance of sanitation, and elucidating the reciprocal relationship between human and animal health in disease control.

###  2. Malaria and NTDs in eg, Zanzibar^9^ and Ethiopia^10^

 Zanzibar’s health authorities, in collaboration with relevant partners and stakeholders, have aligned their malaria and NTD programs through joint planning sessions and common intervention strategies. These included integrated mass drug administration campaigns to address malaria and specific NTDs in targeted areas with the help of different sectors, community health education programs to raise awareness about the prevention and treatment of both malaria and NTDs and the delivery of integrated services at the community level by community health workers, environmental health workers and community volunteers. A coordinated supply chain system, under the government’s control, has been established to manage the procurement, storage, and distribution of drugs and medical supplies for both malaria and NTDs. An integrated Health Management Information Systems has been implemented to collect and analyze health data from both malaria and NTD programs for informed decision-making through Health Management Information Systems reports, disease surveillance data, and epidemiological profiles. Healthcare workers were trained to enhance their skills in diagnosing, treating, and monitoring both malaria and NTD cases. Success factors include alignment with Zanzibar’s broader health policies and strategies through policy documents, strategic plans, and reports on policy implementation and coordination of the initiative through the multisectoral one health approach under the Second Vice President’s Office, demonstrating political will.

 Another notable example is Ethiopia’s development of a cadre of community health extension workers over the past two decades. Initially established to strengthen the national malaria response, this workforce was soon leveraged to deliver integrated, multi-disease services, including for NTDs. This reform, driven by strong leadership, was supported by harmonized domestic and partner investments, including the Global Fund.

###  3. Integration at Multiple Levels: Leprosy in Ghana

 In Ghana, a comprehensive policy framework has been developed to align leprosy control efforts with overarching national health policies and strategies. Coordination mechanisms have been established with other programs, including agriculture, gender, and WASH (water, sanitation, and hygiene). The programs integrate leprosy awareness and services into existing community health structures, healthcare workers undergo training on the diagnosis, treatment, and social aspects of leprosy and other NTDs and data for leprosy and certain other NTDs are being incorporated into the national health information and reporting systems. These efforts allow synchronization of activities, promote resource-sharing, foster collaboration among diverse health initiatives and facilitate enhanced monitoring and evaluation. Advocacy efforts are also aligned: integrating awareness campaigns into general health promotion activities fosters understanding that leprosy and NTDs are treatable conditions and not causes for discrimination.

## Key Actions for Stakeholders for Country Level Integration of Health and Beyond

 This call-to-action advocates for a shift away from fragmented, disease-specific approaches towards integrated approaches, especially for disease control and elimination, through these key actions:

1. *Integrated planning:* Every national and sub-national health or development plan should integrate the relevant NTDs, so that the needs of the poorest communities, who are most affected, are consistently prioritized and synchronized with broader health goals.
2. *Multi-sector coordination:* NTDs should be a standing agenda item in health partner coordination meetings, with the inclusion of WASH (water, sanitation, and hygiene), vector control, and One Health stakeholders.
3. *Incentivise integration:* Funders of health programs in low- and middle-income countries should invest in integrated health initiatives that align with domestic programming. This demands frameworks for multi-disease, cross-sectoral, and systems-centric investments that allow monitoring and attribution, to answer the demands of funders’ constituencies.
4. *Learning by doing:* Implementation research should continue to accompany integration efforts, without delaying it - building on what is already known and driving immediate and practical action.
5. *Consolidate and promote “integration essentials”:* Work across initiatives and programs to establish a small set of “Integration Essentials,” based on existing recommendations and guidance, that define minimum requirements for integration in various contexts, particularly for funding from global health initiatives.

By breaking down silos and embracing integration as the norm – wherever and whenever it makes sense - we can better serve the communities, health workers, and systems. It is time for global health to evolve beyond fragmented efforts and to make integration a reality. The steps outlined in this paper provide key actions to achieve this goal: let’s not wait to integrate.

## Acknowledgements

 The authors would like to express our sincere gratitude to all members of the Integration Working Group (IWG), for their expert and invaluable contributions to this work. In particular, we want to thank Dr. Paul Cantey for his significant contribution to earlier drafts. The IWG is a platform for country representatives, local and international implementation partners, global health donors, and other stakeholders, to advance cross-disease and cross-sector integration through thought leadership and collective analysis of experience and available research. IWG is convened by the Global Institute for Disease Elimination. The authors also thank Dr. Ye Min Thet for his contribution to the initial draft, Dr. Fatma M. Kabole, Dr. Benedict Quao and Grace Hameja for the examples, and Wiebke Kobel, Sarah Shultz and Kaitlyn Paltzat for background literature reviews.

## Disclosure of artificial intelligence (AI) use

 Not applicable.

## Ethical issues

 Not applicable.

## Conflicts of interest

 Authors declare that they have no conflicts of interest.

## Disclaimers

 The views expressed herein are those of the authors and do not necessarily reflect the official policy or position of the organizations they are affiliated with.

## Endnotes


^[1]^ One pragmatic definition would be: managerial or operational changes to (health systems to) bring together inputs, delivery, management and organization of particular functions as a means of improving eg, coverage, access, quality, acceptability, and/or (cost)-effectiveness.

## Supplementary files


Supplementary file 1. Information NTD Integration Literature.
